# Aggregation and combination of cardiovascular risk factors and their association with 10-year all-cause mortality: the PERU MIGRANT Study

**DOI:** 10.1186/s12872-021-02405-8

**Published:** 2021-12-07

**Authors:** Janina Bazalar-Palacios, J. Jaime Miranda, Rodrigo M. Carrillo-Larco, Robert H. Gilman, Liam Smeeth, Antonio Bernabe-Ortiz

**Affiliations:** 1 School of Public Health and Administration, Universidad Peruana Cayetano Heredia, Lima, Peru; 2 CRONICAS Center of Excellence in Chronic Diseases, Universidad Peruana Cayetano Heredia, Av. Armendáriz 445, Miraflores, Lima 18, Peru; 3 Department of Medicine, School of Medicine, Universidad Peruana Cayetano Heredia, Lima, Peru; 4 Department of Epidemiology and Biostatistics, School of Public Health, Imperial College London, London, UK; 5 Department of International Health, Bloomberg School of Public Health, Johns Hopkins University, Baltimore, MD USA; 6 Faculty of Epidemiology and Population Health, London School of Hygiene and Tropical Medicine, London, UK

**Keywords:** Survival, Comorbidity, Prospective studies (MeSH NLM)

## Abstract

**Objective:**

To estimate the association between the aggregation and pair-wise combination of selected cardiovascular risk factors (CVRF) and 10-year all-cause mortality.

**Methods:**

Secondary data analysis of the PERU MIGRANT study, a prospective population-based cohort. Ten-year all-cause mortality was determined for participants originally enrolled in the PERU MIGRANT Study (baseline in 2007) through the National Registry of Identification and Civil Status. The CVRF included hypertension, type 2 diabetes mellitus, hypercholesterolemia, and overweight/obesity. Exposures were composed of both the aggregation of the selected CVRF (one, two, and three or more CVRF) and pair-wise combinations of CVRF. Cox regression models were used to calculate hazard ratios (HR) and 95% confidence intervals (95% CI).

**Findings:**

Of the 989 participants evaluated at baseline, 976 (98.8%) had information about vital status at 10 years of follow-up (9992.63 person-years), and 63 deaths were recorded. In the multivariable model, adjusting for sociodemographic and lifestyle variables, participants with two CVRF (HR: 2.48, 95% CI: 1.03–5.99), and those with three or more CVRF (HR: 3.93, 95% CI: 1.21–12.74) had higher all-cause mortality risk, compared to those without any CVRF. The pair-wise combinations associated with the highest risk of all-cause mortality, compared to those without such comorbidities, were hypertension with type 2 diabetes (HR: 11.67, 95% CI: 3.67–37.10), and hypertension with overweight/obesity (HR: 2.76, 95% CI: 1.18–6.71).

**Conclusions:**

The aggregation of two or more CVRF and the combination of hypertension with type 2 diabetes or overweight/obesity were associated with an increased risk of 10-year all-cause mortality. These risk profiles will inform primary and secondary prevention strategies to delay mortality from cardiovascular risk factors.

**Supplementary Information:**

The online version contains supplementary material available at 10.1186/s12872-021-02405-8.

## Introduction

Cardiovascular diseases represent the main cause of mortality worldwide. Of the total (17.7 million) estimated deaths worldwide from cardiovascular disease, more than three-quarters occurred in low- and middle-income countries (LMICs) [1]. These death rates are mainly driven by the presence of different cardiovascular risk factors, including high blood pressure, type 2 diabetes mellitus, obesity, and hypercholesterolemia [2]. However, in many cases, these cardiovascular risk factors are not in isolation, but aggregated with other chronic conditions, in a way that increases the risk of disability, development of cardiovascular events, and mortality.

Multimorbidity, defined as the presence of two or more chronic conditions without any one disease taking priority over the other, is still more common in high-income countries (HICs). Previous research conducted in developed countries found that the risk of mortality is higher among individuals with more than two diseases compared to disease-free individuals or those with one disease [3, 4]. In low- and middle-income countries, the risk of mortality due to multimorbidity is rapidly increasing [3], including Peru, where the prevalence of cardiovascular risk factors are in the rise [6, 7]. Despite the burden of multimorbidity in LMICs, the characterization and long-term consequences of having multiple chronic conditions remain largely unknown. As a result, medical care in LMICs often focuses on treating one disease rather than multiple diseases at once.

Current evidence evaluates the potential effect of multimorbidity on disability and mortality, but do not determine which of the risk factors or chronic conditions are really associated with important outcomes. Evaluating which combination of conditions occurs more frequently and is associated with worse outcomes, such as mortality, should be particularly important in LMICs. Understanding which higher-risk combinations should be target will help allocate resources more efficiently, especially in resource-constrained settings. Although some cohort studies have explored which are the most prevalent and lethal combinations of chronic conditions, one of them explored two to five diseases [8], whereas another one explored two and three conditions [9]. Moreover, the first study included individuals aged ≥ 65 years and the second study included a limited sample of individuals ≥ 85 years [9]. Thus, multimorbidity is not only a problem for the elderly and in order to intervene efficiently it is necessary to focus on the general population.

Cardiovascular risk factors are associated with lifestyle and have changed rapidly in recent decades, some driven by urbanization [10]. Environmental changes and population flows have led to increased urbanization around the world, especially in low- and middle-income countries. The degree of urbanization is closely related to the distinctive characteristics of the health profile of rural and urban inhabitants, as well as of migrants from rural to urban areas, causing in the latter group a change in their lifestyle. The multimorbidity profile of these migrant groups is largely unknown and needs to be further examined.

Therefore, in order to provide evidence on the long-term effects of multimorbidity in LMICs, we aimed to estimate the 10-year all-cause mortality risk associated with the aggregation of selected cardiovascular risk factors. In addition, we also evaluated the strength and magnitude of the association between each pair-wise combination of these cardiovascular risk factors and the all-cause mortality risk.

## Materials and methods

### Study design

The PERU MIGRANT Study is a prospective population-based cohort [4], that was established to assess the risk factors of cardiovascular disease in three population groups in Peru: urban dwellers, who were born and currently live in Pampas de San Juan de Miraflores, a periurban area in Lima; rural inhabitants from San José de Secce and Chacas in Ayacucho (highlands settings); and rural-to-urban migrants, inhabitants born in Ayacucho (rural) who migrated to Pampas de San Juan de Miraflores (urban).

### Participants

Participants were recruited from 2007 to 2008 using a random sampling technique stratified by sex and age (30–39, 40–49, 50–59 and 60 + years) in each population group using household census data [4]. For all study groups, men and women ≥ 30 years of age and habitual residents (≥ 6 months) of the study area were considered eligible. Pregnant women and people with mental disorders that prevented them from giving informed consent were excluded.

Following a random selection process, participants were invited to participate. They provided consent to participate in the study, completed a questionnaire, and had their anthropometric measurements assessed [4]. Survival data were retrieved through the National Registry of Identification and Civil Status (RENIEC) information to ascertain vital status and date of death (or censoring) when relevant.

### Variables

#### Outcome

All-cause mortality at 10 years of follow-up was the outcome of interest. After the RENIEC database was searched, participants were classified as alive or dead. Those participants who were not available in that database (i.e. their national identity number was incorrectly recorded), were considered censored at the last time of contact (i.e. first or second in-person follow-up) [5, 6].

#### Independent variables

Aggregation and pair-wise combinations of cardiovascular risk factors were the independent variables. Risk factors included hypertension, type 2 diabetes mellitus, hypercholesterolemia, and overweight/obesity. These chronic cardiovascular risk factors were studied at baseline following standard procedures for all participants [4]. Hypertension was defined as systolic blood pressure (SBP) ≥ 140 mm Hg, diastolic blood pressure (DBP) ≥ 90 mm Hg or self-reported diagnosis with the use of anti-hypertensive medications [7]. Type 2 diabetes mellitus was defined based on fasting blood glucose ≥ 126 mg/dl or self-reported diagnosis with the use of anti-diabetic medications [8]. Overweight/obesity was defined as a body mass index (BMI) ≥ 25 kg/m^2^ [9]. Hypercholesterolemia was defined as total cholesterol ≥ 200 mg/dl [10], and was assessed from blood samples drawn after an 8-h fasting period.

We examined mortality rates with different cardiovascular risk profiles. In the *first part,* cardiovascular risk factors were aggregated and then split into one, two, and three or more cardiovascular risk factors. On the other hand, the *second part* comprised six groups of analysis with selected cardiovascular risk factors (e.g., group 1: hypertension with type 2 diabetes mellitus; group 2: hypertension with hypercholesterolemia, etc.) Each group was evaluated in four categories: disease-free individuals (reference), people with only one disease (e.g., only hypertension and only type 2 diabetes mellitus), and the pair-wise combination (e.g., hypertension with type 2 diabetes mellitus). The rationale of using this approach was to capture the individual effect of each chronic condition in addition with the pair-wise combination using the same model, and in this way, avoiding misclassification bias.

#### Covariables

Other variables were included in the analysis as potential confounders. *Sociodemographic variables* included age (30–39, 40–49, 50–59, and 60 + years), sex, migrant status (rural, migrant or urban), education level (< 7 years of education vs. ≥ 7 years of education), and socioeconomic status (low, medium, or high), measured using a wealth index based on household income, assets and household facilities. *Lifestyle variables* included tobacco use (having smoked at least one cigarette per day compared to never users in the 6 months prior to the interview, and classified as yes or no), alcohol consumption (self-reported consumption of ≥ 6 beers or its equivalent in alcohol with other beverages on the same occasion at least once a month, and classified into low or high), HDL cholesterol (> HDL-c 40 mg/dl in men and > 50 mg/dl in women) [10], and high waist circumference (> 80 cm in women and > 90 cm in men) [11].

### Statistical analysis

The statistical analysis was conducted in STATA 14 for Windows (STATA Corp, College Station, TX, USA). The characteristics of the study population were tabulated according to the population group at baseline, and Chi-squared tests were used to compare categorical variables.

For the bivariate analysis, the log rank test was used to evaluate the association between sociodemographic variables, lifestyles, each cardiovascular risk factor, and aggregated cardiovascular risk factors and all-cause mortality. The Cox proportional hazard model was used to estimate the effect of the aggregation of cardiovascular risk factors on all-cause mortality. Crude and adjusted models were computed. Adjusted models included sociodemographic and lifestyle variables.

The association between the pair-wise combinations of cardiovascular risk factors and all-cause mortality were evaluated separately using crude and adjusted Cox regression models. Adjusted models included sociodemographic and lifestyles variables, as well as the cardiovascular risk factors not included in the pair-wise combination. Cox regression assumptions of independent observations, and independent censorship of survival and proportional risks were assessed. The latter assumption was evaluated using the global test of Schoenfeld residuals. Given the number of confounding variables, the variance inflation factor was also utilized to evaluate the presence of collinearity.

## Results

### Characteristics of the study population

We analyzed the data of 989 participants included at baseline. Mean age was 47.9 years (SD: 12.0), and 52.8% of the cohort was female. Regarding population groups, 201 (20.3%), 589 (59.6%), and 199 (20.1%) were rural, rural-to-urban migrants, and urban participants, respectively (Table 1).

**Table 1:** Baseline characteristics of the PERU MIGRANT Study migrant status

Characteristics	Rural N = 201	Migrant N = 589	Urban N = 199	p*
*Sex*				0.951
Female	106 (52.7)	309 (52.5)	107 (53.8)	
Male	95 (47.3)	280 (47.5)	92 (46.2)	
*Age*				0.574
30–39 years	61 (30.3)	164 (27.8)	57 (28.6)	
40–49 years	56 (27.9)	173 (29.4)	53 (26.6)	
50–59 years	46 (22.9)	167 (28.4)	59 (29.6)	
60 + years	38 (18.9)	85 (14.4)	30 (15.1)	
*Education level*				< 0.001
< 7 years of education	162 (80.6)	282 (48.0)	36 (18.2)	
> 7 years of education	39 (19.4)	306 (52.0)	162 (81.8)	
*Asset index*				< 0.001
Lowest	124 (61.7)	242 (41.1)	67 (33.7)	
Middle	14 (7.0)	156 (26.5)	69 (34.7)	
Highest	63 (31.3)	191 (32.4)	63 (31.7)	
*Current smoker*				< 0.001
No	190 (94.5)	530 (90.0)	159 (79.9)	
Yes	11 (5.5)	59 (10.0)	40 (20.1)	
*Alcohol consumption*				0.401
Low	178 (88.6)	542 (92.0)	182 (91.5)	
High	23 (11.4)	47 (8.0)	17 (8.5)	
*Waist circumference*				< 0.001
Normal	168 (84.8)	233 (39.7)	66 (33.3)	
High	30 (15.2)	354 (60.3)	132 (66.7)	
*HDL-c levels*				0.735
Normal	113 (56.2)	341 (57.9)	108 (54.3)	
High	88 (43.8)	248 (42.1)	91 (45.7)	
*Diabetes mellitus*				0.002
No	197 (98.5)	568 (96.4)	183 (92.0)	
Yes	3 (1.5)	21 (3.6)	16 (8.0)	
*Total cholesterol*				< 0.001
Normal	186 (92.5)	368 (62.6)	128 (64.3)	
High	15 (7.5)	220 (37.4)	71 (35.7)	
*Hypertension*				< 0.001
No	177 (88.1)	518 (88.1)	144 (72.4)	
Yes	24 (11.9)	70 (11.9)	55 (27.6)	
*Overweight/obesity*				< 0.001
Normal	160 (80.4)	189 (32.4)	56 (28.4)	
Overweight/obesity	39 (19.6)	395 (67.6)	141 (71.6)	
*Aggregation of CVRF*				< 0.001
No CVRF	133 (67.2)	124 (21.3)	37 (18.8)	
One CVRF	51 (25.8)	256 (43.9)	68 (34.5)	
Two CVRF	14 (7.1)	165 (28.3)	64 (32.5)	
Three or more CVRF	0 (0.0)	38 (6.5)	28 (14.2)	

The entries in parentheses refer to the corresponding percentages (%)

CVRF, Cardiovascular Risk Factors

*Chi-squared test of homogeneity

### Prevalence of multimorbidity at baseline and 10-year all-cause mortality

At baseline, the prevalence of multimorbidity was 31.6% (95% CI: 28.7%–34.6%). Two cardiovascular risk factors were present in 25.0% (95% CI: 22.4%–27.8%) of the study population. Three or more risk factors were present in 6.6% (95% CI: 5.3%–8.5%). These estimates were higher in the urban group than in the other two population groups (Table 1)*.*

At 10-years of follow-up, the mortality incidence of 976 (98.8%; 9992.6 person-years) participants was assessed. There were 63 deaths, resulting in an overall mortality rate of 6.3 (95% CI: 4.9–8.1) per 1,000 person-years, distributed as follows: 9.1 (95% CI: 5.7–14.4) in the rural group, 6.4 (95% CI: 3.7–11.1) in the urban group, and 5.3 (95% CI: 3.8–7.5) in the rural to urban migrant group. Male sex, older age, low education level, low asset index, high waist circumference, type 2 diabetes, and hypertension were characteristics associated with higher all-cause mortality (Additional file 1: Table S1).

### Aggregation and pair-wise combinations of cardiovascular risk factors and 10-year all-cause mortality

The population characteristics according to vital status are shown in Additional file 1: Table S1). In the multivariate models adjusting for sociodemographic and lifestyle variables, participants with two cardiovascular risk factors had more than twice all-cause mortality risk compared to those without cardiovascular risk factors. Similarly, people with three or more cardiovascular risk factors had almost four times higher all-cause mortality risk than those without any cardiovascular risk factors (Table 2 & Additional file 1: Table S2).

**Table 2:** Effect of the aggregation of cardiovascular risk factors and 10-year mortality

Variables	Crude model		Adjusted model*	
	HR (95% IC)	p-value	HR (95% IC)	p-value
*Aggregation of CVRF*				
No CVRF	Ref.		Ref.	
One CVRF	1.50 (0.75–3.02)	0.252	2.04 (0.96–4.33)	0.064
Two CVRF	1.71 (0.82–3.58)	0.155	2.48 (1.03–5.99)	0.043
Three or more CVRF	2.27 (0.85–6.05)	0.101	3.93 (1.21–12.74)	0.023

HR, hazard ratio; IC, confidence interval; CVRF, Cardiovascular Risk Factors

*Adjusted by sociodemographic variables (age, sex, migrant status, level of education and asset index) and lifestyle variables (current smoker, alcohol consumption, HDL-c levels, and waist circumference)

When pair-wise combinations were assessed, subjects with hypertension and type 2 diabetes had a 12-fold increase in the risk of all-cause mortality compared to those without any of these conditions. In the same model, hypertension alone doubled such risk. On the other hand, subjects with hypertension and overweight/obesity together had almost three times the risk of all-cause mortality, whereas hypertension alone increased such risk on 3.2 times (Table 3 & Additional file 1: Table S3).

**Table 3:** Pair-wise combinations of cardiovascular risk factors and 10-year mortality

Variables	Crude model	Adjusted model
	HR (IC 95%)	HR (IC 95%)
*Hypertension + Type 2 diabetes mellitus (n = 962) *		
Normal	Ref.	Ref.
Hypertension	5.08 (3.01–8.58)	2.12 (1.17–3.85)
Type 2 Diabetes mellitus	1.92 (0.46–8.02)	1.30 (0.29–5.77)
Hypertension + Type 2 Diabetes mellitus	9.29 (3.28–26.27)	11.67 (3.67–37.10)
*Hypertension + Hypercholesterolemia (n = 961)*		
Normal	Ref.	Ref.
Hypertension	6.88 (3.90–12.13)	2.51 (1.32–4.75)
Hypercholesterolemia	0.75 (0.34–1.67)	0.80 (0.34–1.87)
Hypertension + Hypercholesterolemia	2.46 (1.01–6.00)	2.00 (0.76–5.28)
*Hypercholesterolemia + Type 2 Diabetes mellitus (n = 961)*		
Normal	Ref.	Ref.
Hypercholesterolemia	0.62 (0.32–1.16)	0.86 (0.42–1.77)
Type 2 Diabetes mellitus	3.41 (1.22–9.48)	3.38 (1.10–10.37)
Hypercholesterolemia + Type 2 Diabetes mellitus	1.47 (0.36–6.06)	1.61 (0.36–7.15)
*Hypertension + Overweight/obesity (n = 964)*		
Normal	Ref.	Ref.
Hypertension	9.45 (4.67–19.11)	3.19 (1.48–6.86)
Overweight/obesity	0.89 (0.45–1.77)	1.12 (0.45–2.77)
Hypertension + Overweight/obesity	3.23 (1.54–6.80)	2.76 (1.18–6.71)
*Overweight/obesity + Hypercholesterolemia (n = 962)*		
Normal	Ref.	Ref.
Overweight/obesity	0.91 (0.51–1.64)	1.37 (0.58–3.23)
Hypercholesterolemia	0.97 (0.37–2.56)	1.41 (0.49–4.04)
Overweight/obesity + Hypercholesterolemia	0.54 (0.25–1.16)	0.76 (0.26–2.25)
*Overweight/obesity + Type 2 Diabetes mellitus (n = 961)*		
Normal	Ref.	Ref.
Overweight/obesity	0.74 (0.43–1.27)	1.17 (0.51–2.65)
Type 2 Diabetes mellitus	4.48 (1.06–18.87)	6.00 (1.33–27.11)
Overweight/obesity + Type 2 Diabetes mellitus	1.95 (0.68–5.58)	2.34 (0.66–8.36)

## Discussion

In our study conducted in a low-resource LMIC setting, we found that several aggregation or combination patterns of cardiovascular risk factors were strongly associated with an increased 10-year all-cause mortality risk. Of note, the combination of hypertension and type 2 diabetes mellitus had a tenfold higher risk of mortality, whereas hypertension and overweight/obesity increased the 10-year all-cause mortality risk up to nearly four-fold. Additionally, having two or more cardiovascular risk factors was associated with a greater risk of mortality.

Previous studies have examined the risk of mortality among individuals with two or more chronic conditions compared to those who were free of disease. Three independent conditions, i.e., diabetes mellitus, stroke, and myocardial infarction, have been associated with a similar increase in the risk of mortality, but their aggregation had a multiplicative effect on the risk of death [12]. Another recent study found that the risk of mortality associated with more than four chronic conditions was the same for each condition evaluated individually [13]. Our study, despite having evaluated a limited number of cardiovascular conditions, shows an additive effect for each additional cardiovascular risk factor as well as varying additive effects with different conditions. The effect of the aggregation of cardiovascular factors risk on mortality risk may be explained by the duration and severity of each additional risk factor [14].

We found that the co-occurrence of several pairs of diseases had a multiplicative effect on the all-cause mortality risk. Hypertension-type 2 diabetes mellitus was associated with a twelve-fold higher all-cause mortality risk compared to a nearly three-fold risk in the case of hypertension and overweight/obesity. Our estimates were similar to those reported by other studies, in which cardiovascular mortality was elevated among individuals with type 2 diabetes mellitus and hypertension [15–17]. However, our findings differ with those reported by a meta-analysis, which demonstrated that high systolic blood pressure plus high serum cholesterol substantially increased cardiovascular mortality risk [18]. In this study, hypercholesterolemia, and its co-occurrence with type 2 diabetes mellitus, overweight/obesity and hypertension had a minimal effect. Perhaps these comorbidities attenuated the proportional effects of hypercholesterolemia on the risk of mortality. Our results also showed a non-significant association between overweight/obesity and type 2 diabetes mellitus and mortality risk. Additionally, hypertension was associated with a higher mortality risk alone compared to when it co-occurred with certain cardiovascular risk factors (e.g., hypertension with overweight/obesity and hypertension with hypercholesterolemia). However, when hypertension was combined with type 2 diabetes mellitus, it resulted in a multiplicative mortality risk. The consistency of our findings has been reported in other studies [19, 20]. A possible explanation for this finding is that inadequate control of hypertension and diabetes increases the likelihood of life-threatening complications and mortality. Therefore, the data presented highlights the need for primary prevention of both hypertension and the combination of hypertension with other cardiovascular risk factors.

The results of the prevalence of multiple conditions in LMICs vary. In this study, we evaluated the four most common chronic conditions in LMICs. Despite the limited number of chronic conditions chosen, the overall proportion of people with multimorbidity was high (25%), especially among urban dwellers. Our estimates of multimorbidity were similar to those reported in a study conducted in six LMICs [21], as well as others carried out in Scotland [22], and the Netherlands [23, 24]. However, the prevalence of multimorbidity was lower compared to a recent study that included nine HICs. In this study, the authors used 12 of the most prevalent chronic conditions, and the data collection was performed between 2007 and 2012. The highest frequency of multimorbidity was observed in Russia (71.9%) and the lowest in China (45.1%) [25]. The variability in multimorbidity between studies can be attributed in part to the definition of multimorbidity used in each study, as there is a lack of consensus regarding it. The Academy of Medical Sciences has suggested using “the presence of two or more chronic conditions without any one disease taking priority over the other” [3], and our analysis has applied that definition, but only for four conditions. Differences could also be due to the number of conditions included in that definition; socioeconomic differences resulting in lifestyle changes (e.g. improved transit that leads to less physical activity), sociodemographic differences between countries such as age and sex distribution, and the country’s phase of epidemiological and nutritional transition (e.g., greater exposure to fast foods) [25–28]. Due to this, it is necessary to conduct country-specific studies to better characterize and understand the epidemiology of multimorbidity. Currently, the provision of medical care in many LMIC settings is largely based on the treatment of individual diseases. Additionally, although there are available guidelines for the treatment of people with multiple morbidities [29], it is necessary that these guidelines include care and treatment for people with LMIC-specific multimorbidity [30].

One of the main strengths of this study is the 10-year follow-up in a LMIC setting and the use of a national records (RENIEC) database to obtain information about the number and date of deaths, thereby decreasing the differential classification bias. Additionally, participants were evaluated with standardized instruments and procedures. Also, lost to follow up rates were very low (i.e., ​​1·2% of the total study population). Finally, we reduced misclassification bias by using four categories for each pair-wise combination instead of using only two (having or nor having the two conditions).

This study also has limitations. The limited number of deaths, despite the 10 years of follow-up, provides point estimates with wide confidence intervals. In addition, only four chronic cardiovascular conditions (hypertension, type 2 diabetes, hypercholesterolemia, and overweight/obesity) were evaluated at baseline, which limits a more detailed exploration of the potential effect of other conditions on mortality. Moreover, new cases of hypertension, type 2 diabetes or hypercholesterolemia, or the variation of BMI over time was not considered. Besides, cardiovascular death should have been ideally the outcome of interest. However, specific cause of death was not available. Finally, our biostatistical models only adjusted for main sociodemographic and lifestyle factors as confounding variables. We did not include other factors, such as race, in our baseline analysis.

## Conclusion

After a 10-year follow-up period, we observed a higher risk of all-cause mortality when more cardiovascular risk factors were present. Additionally, the pair-wise combinations of hypertension and type 2 diabetes mellitus and hypertension and overweight/obesity had the highest risks of death. These results suggest the need for better interventions targeted at people with multiple chronic conditions in LMIC settings.

## Supplementary Information

**Additional file 1:** Aggregation and pair-wise combinations of cardiovascular risk factor and 10-year all-cause mortality.

## Data Availability

The datasets used for this study are available from the corresponding author.
