# Supplementary material for: Aggregation and combination of cardiovascular risk factors and their association with 10-year all-cause mortality: the PERU MIGRANT Study
Source: BMC Cardiovasc Disord. 2021 Dec 7;21:582. doi: 10.1186/s12872-021-02405-8 (PMC8650245; doi:10.1186/s12872-021-02405-8)
Supplement: Supplementary file 1 — Additional file 1: Aggregation and pair-wise combinations of cardiovascular risk factor and 10-year all-cause mortality. [file 12872_2021_2405_MOESM1_ESM.docx]

Aggregation and combination of cardiovascular risk factors and their association with 10-year all-cause mortality: The PERU MIGRANT Study

# Supplementary Material

## Supplementary Table 1: Factors associated with 10-year all-cause mortality

| **Variables** | | **Vital status** | | **p*** |
| --- | --- | --- | --- | --- |
|  |  | **Alive (n=913)** | **Dead (n=63)** |  |
| ***Sex*** | |  |  | 0.004 |
|  | Female | 491 (95.7) | 22 (4.3) |  |
|  | Male | 422 (91.1) | 41 (8.9) |  |
| ***Age*** | |  |  | <0.001 |
|  | 30-39 years | 279 (99.3) | 2 (0.7) |  |
|  | 40-49 years | 272 (97.8) | 6 (2.2) |  |
|  | 50-59 years | 254 (94.1) | 16 (5.9) |  |
|  | 60+ years | 108 (73.5) | 39 (26.5) |  |
| ***Education level*** | |  |  | 0.001 |
|  | <7 years of education | 428 (90.9) | 43 (9.1) |  |
|  | >7 years of education | 483 (96.0) | 20 (4.0) |  |
| ***Asset index*** | |  |  | 0.012 |
|  | Lowest | 386 (91.0) | 38 (9.0) |  |
|  | Middle | 229 (96.6) | 8 (3.4) |  |
|  | Highest | 298 (94.6) | 17 (5.4) |  |
| ***Migrant status*** | |  |  | 0.193 |
|  | Rural | 178 (90.8) | 18 (9.2) |  |
|  | Migrant | 550 (94.5) | 32 (5.5) |  |
|  | Urban | 185 (93.4) | 13 (6.6) |  |
| ***Current smoker*** | |  |  | 0.688 |
|  | No | 811 (93.6) | 55 (6.4) |  |
|  | Yes | 102 (92.7) | 8 (7.3) |  |
| ***Alcohol consumption*** | |  |  | 0.521 |
|  | Low | 834 (93.7) | 56 (6.3) |  |
|  | High | 79 (91.9) | 7 (8.1) |  |
| ***Waist circumference*** | |  |  | 0.032 |
|  | Normal | 422 (91.7) | 38 (8.3) |  |
|  | High | 486 (95.1) | 25 (4.9) |  |
| ***HDL-c levels*** | |  |  | 0.814 |
|  | Normal | 520 (93.7) | 35 (6.3) |  |
|  | High | 393 (93.3) | 28 (6.7) |  |
| ***Total cholesterol*** | |  |  | 0.128 |
|  | Normal | 623 (92.8) | 48 (7.2) |  |
|  | High | 290 (95.4) | 14 (4.6) |  |
| ***Diabetes mellitus*** | |  |  | 0.019 |
|  | No | 879 (93.9) | 57 (6.1) |  |
|  | Yes | 33 (84.6) | 6 (15.4) |  |
| ***Overweight/obesity*** | |  |  | 0.298 |
|  | Normal | 371 (93.0) | 28 (7.0) |  |
|  | Overweight and obese | 540 (94.6) | 31 (5.4) |  |
| ***Hypertension*** | |  |  | <0.001 |
|  | No | 795 (95.9) | 34 (4.1) |  |
|  | Yes | 118 (80.3) | 29 (19.7) |  |
| ***Aggregation of CVRF*** | |  |  | 0.336 |
|  | No CVRF | 277 (95.8) | 12 (4.2) |  |
|  | One CVRF | 349 (93.8) | 23 (6.2) |  |
|  | Two CVRF | 225 (93.0) | 17 (7.0) |  |
|  | Three or more CVRF | 59 (90.8) | 6 (9.2) |  |
| Abbreviations: CVRF, Cardiovascular Risk Factors.  *Log rank test for equality of survivor functions. | | | | |
| The entries in parentheses refer to the corresponding percentages (%). | | | | |

## Supplementary Table 2: Factors independently associated with 10-year mortality.

| **Variables** | | **Crude model** | | **Adjusted model*** | | |
| --- | --- | --- | --- | --- | --- | --- |
|  |  | **HR (95% IC)** | **p-value** | | **HR (95% IC)** | **p-value** |
| Sex | |  |  | |  |  |
|  | Female | Ref. |  | | Ref. |  |
|  | Male | 2.12 (1.26 - 3.56) | 0.005 | | 2.64 (1.41 – 4.95) | 0.002 |
| Age (years) | |  |  | |  |  |
|  | 30-39 years | Ref. |  | | Ref. |  |
|  | 40-49 years | 3.03 (0.61 – 15.04) | 0.174 | | 2.63 (0.53 – 13.16) | 0.237 |
|  | 50-59 years | 8.52 (1.96 – 37.07) | 0.004 | | 6.94 (1.55 – 30.98) | 0.011 |
|  | 60+ years | 43.29 (10.45 – 179.30) | <0.001 | | 31.01 (7.10 – 135.37) | <0.001 |
| Education level | |  |  | |  |  |
|  | <7 years of education | Ref. |  | | Ref. |  |
|  | >7 years of education | 0.42 (0.25 - 0.72) | 0.002 | | 0.77 (0.38 – 1.55) | 0.469 |
| Asset index | |  |  | |  |  |
|  | Lowest | Ref. |  | | Ref. |  |
|  | Middle | 0.37 (0.17 - 0.78) | 0.010 | | 0.43 (0.18 – 1.02) | 0.055 |
|  | Highest | 0.59 (0.33 - 1.04) | 0.069 | | 0.67 (0.37 – 1.24) | 0.209 |
| Migrant status | |  |  | |  |  |
|  | Rural | Ref. |  | | Ref. |  |
|  | Migrant | 0.59 (0.33 - 1.05) | 0.073 | | 0.56 (0.27 – 1.15) | 0.114 |
|  | Urban | 0.71 (0.35 - 1.45) | 0.351 | | 0.82 (0.33 - 2.04) | 0.669 |
| Current smoker | |  |  | |  |  |
|  | No | Ref. |  | | Ref. |  |
|  | Yes | 1.16 (0.55 - 2.44) | 0.688 | | 1.11 (0.49 – 2.52) | 0.802 |
| Alcohol consumption | |  |  | |  |  |
|  | Low | Ref. |  | | Ref. |  |
|  | High | 1.29 (0.59 - 2.84) | 0.522 | | 1.60 (0.68 – 3.75) | 0.278 |
| Waist circumference | |  |  | |  |  |
|  | Normal | Ref. |  | | Ref. |  |
|  | High | 0.58 (0.35 - 0.96) | 0.034 | | 0.64 (0.32 – 1.27) | 0.202 |
| HDL-c levels | |  |  | |  |  |
|  | Normal | Ref. |  | | Ref. |  |
|  | High | 1.06 (0.65 - 1.74) | 0.814 | | 0.62 (0.35 – 1.09) | 0.096 |
| Abbreviation: HR, hazard ratio; IC, confidence interval. | | | | | | |
| *Adjusted by sociodemographic variables (age, sex, migrant status, level of education and asset index) and lifestyle variables (current smoker, alcohol consumption, HDL-c levels, and waist circumference). | | | | | | |

## Supplementary Table 3: Prevalence of pair-wise combinations of cardiovascular risk factors.

| **Pair-wise combinations** | | **n (%)** |
| --- | --- | --- |
| ***Hypertension + Type 2 Diabetes mellitus*** | |  |
|  | Normal | 812 (82.2) |
|  | Hypertension | 136 (13.8) |
|  | Type 2 Diabetes mellitus | 27 (2.7) |
|  | Hypertension + Type 2 Diabetes mellitus | 13 (1.3) |
| ***Hypertension + Hypercholesterolemia*** | |  |
|  | Normal | 590 (59.8) |
|  | Hypertension | 91 (9.2) |
|  | Hypercholesterolemia | 248 (25.1) |
|  | Hypertension + Hypercholesterolemia | 58 (5.9) |
| ***Hypercholesterolemia + Type 2 Diabetes mellitus*** | |  |
|  | Normal | 662 (67.1) |
|  | Hypercholesterolemia | 285 (28.9) |
|  | Type 2 Diabetes mellitus | 19 (1.9) |
|  | Hypercholesterolemia + Type 2 Diabetes mellitus | 21 (2.1) |
| ***Hypertension + Overweight/obesity*** | |  |
|  | Normal | 359 (36.5) |
|  | Hypertension | 50 (5.1) |
|  | Overweight/obesity | 476 (48.4) |
|  | Hypertension + Overweight/obesity | 99 (10.0) |
| ***Overweight/obesity + Hypercholesterolemia*** | |  |
|  | Normal | 332 (33.9) |
|  | Overweight/obesity | 342 (34.9) |
|  | Hypercholesterolemia | 73 (7.5) |
|  | Overweight/obesity + Hypercholesterolemia | 233 (23.7) |
| ***Overweight/obesity + Type 2 Diabetes mellitus*** | |  |
|  | Normal | 396 (40.4) |
|  | Overweight/obesity | 543 (55.5) |
|  | Type 2 Diabetes mellitus | 8 (0.8) |
|  | Overweight/obesity + Type 2 Diabetes mellitus | 32 (3.3) |
